# Supplementary material for: Mental health and wellbeing outcomes of youth participation: A scoping review protocol
Source: PLoS One. 2023 Oct 17;18(10):e0293006. doi: 10.1371/journal.pone.0293006 (PMC10581453; doi:10.1371/journal.pone.0293006)
Supplement: S2 Table — (DOCX) [file pone.0293006.s002.docx]

### S2 Table: Preliminary database search strategy

Database: APA PsycINFO

Search conducted: July 2022

| **Search** | **Terms** | **Records retrieved** |
| --- | --- | --- |
| #1 | exp Mental Health/ OR mental health.tw. OR exp Mental Disorders/ OR (mental disorder* or Mental diseas*).tw. OR Anxiety/ or Performance Anxiety/ OR Catastrophizing/ OR (anxiet* or catastrophiz*).tw. OR Depression*.tw. OR suicide/ or attempted suicide/ or suicidal ideation/ OR suicid*.tw. OR psychological distress*.tw. OR empower*.tw. OR exp Hope/ OR hope*.tw. OR confiden*.tw. OR self-concept/ OR Body Image/ OR Self-Efficacy/ OR Self-Compassion/ OR (self concept* or body image* or self efficac* or self compassion*).tw. OR Self-Esteem/ OR Well Being/ OR (self esteem* or self-esteem* or Long?Term Happiness or wellbeing* or well-being*).tw. | 1633397 |
| #2 | leadership/ OR leadership*.mp. OR decision making/ OR decision mak*.mp. OR Policy Making/ OR advoca*.tw. OR civic engageme*.tw.  OR (policy mak* or policy-mak* or advisory committee* or advisory board* or advisory group* or advisory structure* or steering committee* or steering group*).tw. OR Agenc*.tw. OR Political Participation/ OR politics/ OR Activism/ OR (Politic* or stakeholder engagement* or stakeholder participation*).tw. OR Activist*.tw. | 468787 |
| #3 | #1 AND #2 | 114252 |
| #4 | (Young adult participat* or Adolescent Participat* or young people participat* or Youth participat* or Teen* participat* or youth participatory action research or Young adult engagement* or Adolescent engagement* or young people engagement* or Youth engagement* or Teen* engagement*).mp. OR (("young adult*" or "young people*" or adolesc* or youth* or teen*) adj4 (engagement or participat* or "decision?mak*" or "policy?mak*" or leadership* or advocacy or "advisory committee*" or "advisory board*" or "advisory group*" or "advisory structure*" or "steering committee*" or "steering group*" or "user?generat*" or "activis*" or "co?design*" or "codevelop*" or "co?plan*" or "co?evaluat*" or "co?deliver*" or "co?produc*")).tw. OR (("young adult*" or young people* or youth* or teen*) adj3 (Engag* or partcipat* or coproduc* or participatory action* or civic engage*)).tw. | 14053 |
| #5 | (experience* or reflect* or benefit* or perspective* or involv*).tw. | 1658484 |
| #6 | #3 AND #4 | 1233 |
| #7 | #6 AND #5 | 795 |
| Limited to English language literature. No restrictions placed on publication type or date. | | |
