# Supplementary material for: Mental health and wellbeing outcomes of youth participation: A scoping review protocol
Source: PLoS One. 2023 Oct 17;18(10):e0293006. doi: 10.1371/journal.pone.0293006 (PMC10581453; doi:10.1371/journal.pone.0293006)
Supplement: Attachment — Submitted filename: Response to Reviewers.docx [file pone.0293006.s003.docx]

20^th^ April 2023

Dear Emily Chenette,

Thank you for the opportunity to revise and resubmit our manuscript entitled *‘Mental health and wellbeing outcomes of youth participation: A scoping review protocol’*.

We appreciate the time and effort taken by the reviewers and academic editor in providing constructive and insightful feedback on our protocol.

We are pleased to resubmit our revised manuscript for further consideration by PLOS ONE.

Our team have considered and incorporated changes to our manuscript that reflect the academic editor’s and reviewers’ feedback and suggestions. In the following pages, we have provided responses to each point raised by the reviewers, as per your email request.

We hope that our edits and responses below satisfactorily address any issues or concerns raised in the initial peer-review process.

Again, thank you for the opportunity to strengthen our manuscript and for considering our paper as a PLOS ONE publication.

Please let me know if you require any further information and/or clarification.

Kind regards,

Dr Marlee Bower

Research Fellow

The Matilda Centre for Research in Mental Health and Substance Use

Faculty of Medicine and Health, The University of Sydney

+61 2 8627 9332 | [marlee.bower@sydney.edu.au](mailto:marlee.bower@sydney.edu.au) | <https://sydney.edu.au/matilda-centre/>

**Journal Requirements**

1. Please ensure that your manuscript meets PLOS ONE's style requirements, including those for file naming.

*Author response: The author team have updated the manuscript to reflect PLOS ONE’s style requirements, as per the ‘Title, author, affiliations formatting guidelines’ and the ‘Manuscript body formatting guidelines’.*

2. Please confirm that the competing interest statement does not alter your adherence to all PLOS ONE policies on sharing data and materials, by including the following statement: "This does not alter our adherence to PLOS ONE policies on sharing data and materials.” Please include your updated Competing Interests statement in your cover letter; we will change the online submission form on your behalf.

*Author response: The authors confirm that the competing interest statement does not alter adherence to PLOS ONE policies. Please update our competing interest statement to the following:

“MB, ADB, SS and MT are supported by the BHP Foundation. This does not alter our adherence to PLOS ONE policies on sharing data and materials. To our knowledge, remaining authors report no known conflicts of interest for this paper.”*

3. Please review your reference list to ensure that it is complete and correct. If you have cited papers that have been retracted, please include the rationale for doing so in the manuscript text, or remove these references and replace them with relevant current references. Any changes to the reference list should be mentioned in the rebuttal letter that accompanies your revised manuscript. If you need to cite a retracted article, indicate the article’s retracted status in the References list and also include a citation and full reference for the retraction notice.

*Author response: The author team have updated the manuscript to reflect PLOS ONE’s reference style. The authors have downloaded and used the* [*PLOS reference*](https://endnote.com/style_download/plos-public-library-of-science-all-journals/) *style for EndNote.*

**Reviewer’s Comments**

Reviewer #1: Overall this is a well drafted scoping review protocol, and the proposed study is of importance to the youth globally. I have picked a few minor issues for your attention;
1) Line 132, LGBTQIA+ should be written in full for the first time.
2) The scoping review question should capture the population(p), concept (c) and context (c), it seems the context is not stated in your question.
3) Consider presenting the final main search string from your preliminary search - lines 176 & 177.
4) I have noted the involvement of the youth in this study, present ethical clearance reference for this study and explain the selection process for the advisory group.
5) What are the strengths, limitations and de-limitations for the proposed study?
6) Make sure your reference list has document identification numbers (DOI)

*Author response:
1) The acronym ‘LGBTQIA+’ is now written in full in lines 133-134.*

*2) The research questions were developed in accordance with the participant, intervention and outcome (PIO) format, hence the context (C) is not stated, nor is it relevant to our scoping review questions. We have clarified this in lines 172-174, flagging that further information about the study ‘context’ is provided in Stage 3: Evidence selection.*

*3) We thank the reviewer for this suggestion and have made sure to clearly indicate where this is included in the supplementary materials (see lines 202-203). Due to the substantial word count in the final search string, we are unable to include it in the body of the manuscript. However, we have provided a condensed version of the final search terms in lines 194-200.*

*4) Young people were involved in this project in an advisory and co-author capacity (as part of the Youth Mental Health Advisory Team), not as study participants, and hence no ethical clearance or approval is required for their involvement. We have clarified this, as well as details regarding the selection process for the advisory group, remuneration costs and their key responsibilities, in lines 157-170 and lines 292-294.*

*5) Strengths and limitations of the proposed scoping review have now been added to the discussion section in lines 300-316.*

*6) Digital object identifiers (DOIs) have now been added to the reference list.*

Reviewer #2: This is an excellent protocol! I really love that the team is including youth throughout the scoping review process. I have no comments.

*Author response: N/A*
